# Novel Compound Heterozygous *DST* Variants Causing Hereditary Sensory and Autonomic Neuropathies VI in Twins of a Chinese Family

**DOI:** 10.3389/fgene.2020.00492

**Published:** 2020-05-25

**Authors:** Jie-Yuan Jin, Pan-Feng Wu, Ji-Qiang He, Liang-Liang Fan, Zhuang-Zhuang Yuan, Xiao-Yang Pang, Ju-Yu Tang, Li-Yang Zhang

**Affiliations:** ^1^Department of Orthopaedics, Xiangya Hospital of Central South University, Changsha, China; ^2^Department of Neurosurgery, Xiangya Hospital, Central South University, Changsha, China; ^3^School of Life Sciences, Central South University, Changsha, China; ^4^Human Key Laboratory of Animal Models for Human Diseases, School of Life Sciences, Central South University, Changsha, China

**Keywords:** HSAN-VI, *DST*, scoliosis, dystonin, whole-exome sequencing

## Abstract

**Background:** Hereditary sensory and autonomic neuropathies (HSANs) are a rare and severe group of sensory axonal neuropathies. HSANs have been classified into eight groups based on mode of inheritance, clinical features, and the involved genes. HSAN-VI, perhaps the most notable type, is an autosomal recessive disease, which manifests as the severely impaired pain sensitivity, autonomic disturbances, distal myopathy, spontaneous or surgical amputations, and sometimes early death. Mutations in *DST* have been identified as the cause of HSAN-VI. *DST* encodes dystonin, a member of the plakin protein family that is involved in cytoskeletal filament networks. Dystonin has seven major isoforms in nerve, muscle, and epithelium.

**Material and Methods:** The present study investigated a Chinese family with HSAN and explored potential pathogenic variants using whole-exome sequencing (WES). Variants were screened and filtered through bioinformatics analysis and prediction of variant pathogenicity. Co-segregation analysis was subsequently conducted.

**Results:** We identified compound heterozygous variants of *DST* (c.3304G>A, p.V1102I and c.13796G>A, p.R4599H) in two patients.

**Conclusion:** We reported on a Chinese family with HSAN-VI family and detected the disease-causing variants. Our description expands the spectrum of known *DST* variants and contributes to the clinical diagnosis of HSAN-VI.

## Background

Hereditary sensory and autonomic neuropathies (HSANs), also known as hereditary sensory neuropathies (HSNs), are a rare and severe group of sensory axonal neuropathies (Davidson et al., 2012). The cardinal clinical features of HSAN are various sensory and autonomic dysfunctions characterized by the loss of pain and temperature sensations and, in some subtypes, positive sensory symptoms (Leipold et al., 2013). Patients with HSANs might also exhibit altered sweating (hyperhidrosis or anhidrosis), cardiovascular dysregulation, dyskinesia, and gastrointestinal dysmotility (Hepburn et al., 2014; Woods et al., 2015). Other complications include ulcers, infections, osteomyelitis, self-mutilation, and spontaneous or surgical amputations (Phatarakijnirund et al., 2016). HSANs are classified into eight groups based on inheritance, clinical features, and the involved genes (Supplementary Table 1) (Davidson et al., 2012; Heimer et al., 2016; Yozu et al., 2016).

Variants in 18 genes and one locus have been identified as causing HSAN. *DST* was reported to be associated with HSAN-VI (OMIM 614653) in three unrelated families (Edvardson et al., 2012; Manganelli et al., 2017; Fortugno et al., 2019). All HSAN-VI patients presented severely impaired pain sensitivity, autonomic disturbances, and distal myopathy, and some died in early childhood (Edvardson et al., 2012; Manganelli et al., 2017). *DST* is located on 6p12.1 and encodes dystonin, a member of the plakin protein family that is involved in cytoskeletal filament networks (Pool et al., 2005). *DST* has four different promoters and many alternative splicing sites that produce different transcripts, including three major neuronal isoforms (dystonin-a1, a2, and a3), three muscular isoforms (dystonin-b1, b2, and b3), and one epithelial isoform (dystonin-e) (Jefferson et al., 2006; Kunzli et al., 2016). *Dst* variants in mice cause dystonia musculorum (dt), a neurodegenerative disease characterized by progressive degeneration of sensory neurons (Brown et al., 1995).

Here, we report on a family with HSAN-VI in China, in which the HSAN-VI patients harbor compound heterozygosity of *DST* consisting of two missense variants (NM_001144769: c.3304G>A, p.V1102I and c.13796G>A, p.R4599H). These two variants were inherited from the parents. To the best of our knowledge, this combination of variants has not been previously reported.

## Materials and Methods

### Patients and Subjects

The Review Board of Xiangya Hospital of the Central South University approved this research (approval number: 20190038). Written informed consent was obtained from the patients and their guardians, and all subjects consented to participate in this study and to the publication of the images. Blood was collected from the proband and related family members. Segregation analysis was performed for all family members based on whole-exome sequencing (WES) results.

### Quantitative Sensory Testing

Thermal thresholds (cold, warm) were evaluated using a thermal sensory analyzer (TSA 2001, Israel). The base temperature was 32°C, the range was from 0 to 50°C, and the rate of change was 1°C/s. The bilateral hypothenar muscles and dorsum pedis were tested 5 times each at 15 s intervals.

Mechanical pain perception was evaluated using a calibrated monofilament with a bending force of 95 mN that was connected to a sharp non-penetrating probe (50-mm tip). This was applied 10 times for 1–2 s each time. The percentage of stimuli perceived as painful and the pain magnitude using a visual analog scale were recorded. Three null stimuli were randomly applied during testing to evaluate subject reliability (Nolano et al., 2008).

### Whole-Exome Sequencing

Genomic DNA was extracted with a DNeasy blood and tissue kit (Qiagen, Valencia, CA, USA). The Novogene Bioinformatics Institute (Beijing, China) performed exome capture, high-throughput sequencing, and common filtering. All the exomes were captured by means of Agilent SureSelect Human All Exon V5 kits and sequenced with the Illumina HiSeq 2000 platform. After filtering of common variants (allele frequency > 0.05) from the YanHuang genome (http://yh.genomics.org.cn/), Exome Aggregation Consortium (http://exac.broadinstitute.org/), 1000 Genomes Project (https://www.genome.gov/27528684/1000-genomes-project/), Genome Aggregation (http://gnomad.broadinstitule.org), dbSNP (https://www.ncbi.nlm.nih.gov/SNP/), and ESP (http://evs.gs.washington.edu/EVS/) databases; unique single-nucleotide polymorphisms (SNPs); and insertions/deletions (indels) were detected in the subjects. These variants were screened against the list of genes previously identified as being associated with HSAN (Supplementary Table 1) to identify the variants in known causative genes. Then, MutationTaster (http://www.mutationtaster.org/), PolyPhen-2 (http://genetics.bwh.harvard.edu/pph2/), and SIFT (http://provean.jcvi.org/index.php) were used to predict whether the variants affected protein structure and function. Gene function, inheritance pattern, clinical phenotype, and pathogenicity were annotated according to Online Mendelian Inheritance in Man (OMIM) (https://www.omim.org) and American College of Medical Genetics classification (Richards et al., 2015).

### Cosegregation Analysis

Primer pairs were designed via DNASTAR (DST c.3304G>A f: TCAAGATTTCCCGCCTCATAAA, DST c.3304G>A r: GCACACAGATGGGACCATAG; DST c.13796G>A f: CCTGCTTGAAACTGACAGTGTT, DST c.13796G>A r: CCCATATAACATGGTCAGTTTG); the sequences of the primers will be provided upon request. The target fragments were amplified via polymerase chain reaction (PCR) and characterized with an ABI 3100 Genetic Analyzer (ABI, Foster City, CA).

### Total RNA Extraction, RNAseq, and qPCR

Total RNA was extracted using an RNA extraction kit provided by Qiagen (Germany) and then stored at −80°C.

The total RNA was sent to Novogene (Beijing, China) for transcriptome sequencing using the Illumina HiSeq 4000 sequencing platform. Data were compared and analyzed using a high-efficiency combination of node calculations by Dell and stored by Isilon. Genes with a *p* < 0.05, an adjusted *p*-value (padj) < 0.05 and a −1 < log2 fold change > 1 were considered differentially expressed genes.

The total RNA was reverse transcribed using a RevertAid First Strand cDNA Synthesis Kit (Thermo Fisher Scientific, USA) and then subjected to qPCR by 2× SYBR Green qPCR Mix (Thermo Fisher Scientific, USA).

### Data Analysis

Data were processed and graphed using GraphPad Prism 5 software and tested to determine significant differences (*p* < 0.05).

## Case Presentation

### Clinical Features

**Figure 1:**
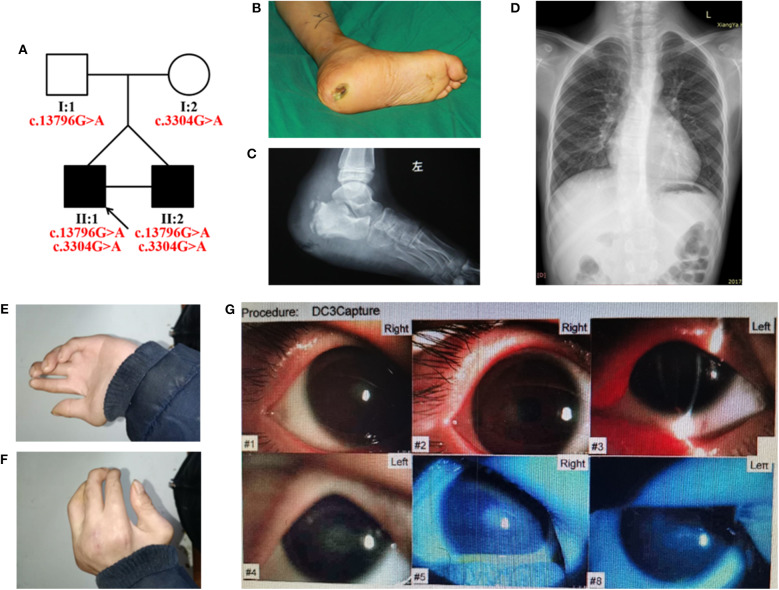
**(A)** Pedigree of the HSAN-VI family with segregation analysis. The black symbols represent the affected members, and the arrow indicates the proband. Red text represents the variants. **(B–G)** Aspects of the phenotype of the proband. The proband has an ankle ulcer **(B)**, necrosis of the astragalus **(C)**, moderate scoliosis **(D)**, phalangeal joint contracture **(E,F)**, and cataract **(G)**.

We identified a Chinese family with HSAN (Figure 1A). The proband (II:1) was admitted to the Department of Orthopedics, Xiangya Hospital of Central South University, for an ulcer near the left ankle and diagnosed with HSAN. The proband was a 14-year-old boy with an identical twin brother (II:2). They were born at term via cesarean section. The height and weight of the proband were 145 cm (<3%) and 26.5 kg (<3%), respectively, without any growth hormone imbalances, and his brother was 150 cm (<3%) tall, and weighed 29.0 kg (<3%), suggesting that they had growth retardation (without hormonal abnormalities).

The proband had severely impaired pain sensitivity (Table 1). He consistently could not perceive noxious thermal/gelid stimuli or innocuous warm stimuli, which had resulted in the ankle ulcer and necrosis of the astragalus (Figures 1B,C). A nerve conduction study revealed peripheral nerve damage in the extremities, especially of the sensory nerves (Supplementary Table 2). The proband had moderate scoliosis (with a Cobb angle of 33°) without centrum malformation (Figure 1D). The younger brother also exhibited HSAN, but his condition was clearly milder. The brothers presented with arthrogryposes of the fingers, foot extroversion, knee recurvation deformities, and myasthenia; hence, they achieved independent ambulation at 3 years of age (Figures 1E,F). The proband had thin blue scleras and corneal ulcers and was subsequently diagnosed with a cataract (Figure 1G). He also displays urinary incontinence. Furthermore, the proband was diagnosed with vascular structure abnormalities via centesis at 18 months of age. Their parents (I:1 and I:2) were unaffected.

**Table 1:** Quantitative sensory testing and deep tendon reflexes.

	**Proband (II:1)**		**II:2**		**Normal values**	
**Temperature sensation thresholds (°C)**	**Cold**	**Warm**	**Cold**	**Warm**	**Cold**	**Warm**
Right hand	15.8	15.4	4.2	4.4	<2.5	<2.1
Left hand	Insensitive	Insensitive	6.2	5.4	<2.5	<2.1
Right foot	Insensitive	Insensitive	8.0	6.8	<4.0	<3.9
Left foot	Insensitive	Insensitive	8.8	8.2	<4.0	<3.9
**Pinprick (% of stimuli perceived as painful)**						
Right hand	10		70		>80	
Left hand	0		20		>80	
Right foot	0		60		>80	
Left foot	0		0		>80	
**Deep tendon reflexes**						
Upper limbs	**+**		**++**		**++**	
Lower limbs	0		0		**++**	

### Genetic Analysis

After filtering the common variants from the 1000 Genomes Project, YH, ExAC, dbSNP132, and ESP databases, 571 unique SNPs were detected in the proband. The variants were filtered against 18 genes known to be involved in HSAN (Supplementary Table 1), and a set of three variants in two genes were identified in the proband (Table 2). By analyzing the bioinformatic predictions, inheritance patterns, OMIM clinical phenotypes, and American College of Medical Genetics classification (Richards et al., 2015) of these three genes, we strongly suspected that variants in *DST* (c.3304G>A, p.V1102I and c.13796G>A, p.R4599H) were the causative genetic variants in these patients.

**Table 2:** Variants identified by WES in combination with HSAN-related gene filtering in the present family.

**Gene**	**Variant**	**Mutation taster**	**PolyPhen-2**	**SIFT**	**1000G**	**ExAC**	**gnomAD**	**AA conservation**	**Type of AA change**	**OMIM clinical phenotype**	**American College of Medical Genetics classification**
*DST*	NM_001144769: c.13796G>A, p.R4599H	D (1.000)	D (1.000)	T (0.136)	0.003	0.00111	0.00115	H	Aliphatic to heterocyclic	AR; Neuropathy, hereditary sensory and autonomic, type VI/AR; Epidermolysis bullosa simplex, autosomal recessive 2	PP3, PP4
*DST*	NM_001144769: c.3304G>A, p.V1102I	D (1.000)	D (0.995)	D (0.000)	–	–	–	H	No change		PM2, PP3
*WNK1*	NM_001184985: c.6613C>T, p.R2205C	D (1.000)	D (1.000)	D (0.000)	0.002	0.0008	0.0004	H	Alkaline to polar	AR; Neuropathy, hereditary sensory and autonomic, type II/AD; Pseudohypoal dosteronism, type IIC	PP3, BS4
*ZFHX2*	NM_033400: c.3676C>T, p.P1226S	D (1.000)	B (0.435)	T (0.378)	–	–	–	M	Nonpolar to polar	AD; ?Marsili syndrome	PM2, BS4, BP4
*ZFHX2*	NM_033400: c.3684_3689del, p.1228_1230del	N (1.000)	**–**	**–**	–	–	–	M	–		PM2, BS4, BP4

*D, disease causing; T, tolerated; B, benign; AA, amino acid; H, highly conserved; M, moderately conserved; AR, autosomal recessive; AD, autosomal dominant*.

**Figure 2:**
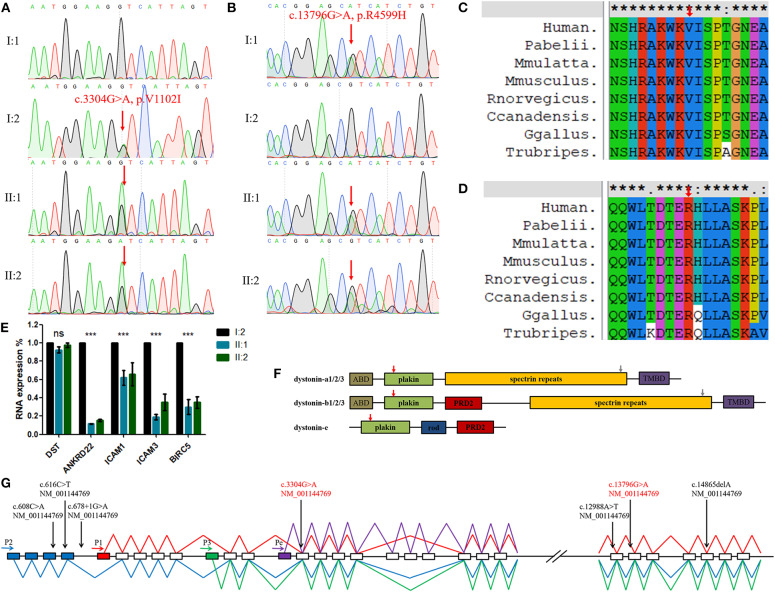
**(A,B)** Sequencing results of the *DST* variants. Sequence chromatograms indicate compound heterozygous variants (c.3304G>A, p.V1102I and c.13796G>A, p.R4599H) in the family with HSAN-VI. **(C,D)** DST peptide sequences surrounding the mutated residues (p.V1102I and p.R4599H) with multiple interspecies alignments generated by ClustalX. The mutation position is indicated with a red arrow. **(E)** The results of qPCR. “***”, statistical significance; “ns”, no statistical significance. **(F)** The structures of three major tissue-specific dystonin isoforms. “ABD”, an actin-binding domain; “plakin”, a plakin domain; “rod”, a rod domain; “PRD2”, the plectin repeats; “spectrin repeats”, spectrin repeats; and “TMBD”, a combination of an EF hand domain and a growth arrest-specific 2 protein-related region. The red (V1102I) and gray (R4599H) arrows indicate the mutated AA site. **(G)** Schematic diagram of *DST* and the variants identified in HSAN-VI patients. Four putative promoters are indicated by horizontal arrows. Blue boxes indicate the unique exons of isoform-2, the red box indicates the unique exon of isoform-1, the green box indicates the unique exon of isoform-3, and the purple box indicates the unique exon of isoform-e. The blue line represents the transcript of isoform-2, the red line represents the transcript of isoform-1, the green line represents the transcript of isoform-3, and the purple line represents the transcript of isoform-e. Full vertical arrows show the positions of the variants of *DST* in the gene. The red text represents the present variants.

Sanger sequencing showed that the *DST* variant (c.3304G>A, p.V1102I) in the identical twins was inherited from their mother and the other (c.13796G>A, p.R4599H) was inherited from their father (Figures 2A,B). This finding indicates that the compound heterozygosity co-segregated within the affected family members. Neither variant was identified in the 200 control cohorts previously studied by our group. The amino acid sequence alignment analysis suggested that these two variants were both located in a highly evolutionarily conserved site (Figures 2C,D).

### RNAseq Analysis

By sequencing the peripheral blood RNA from I:2, II:1, and II:2 and excluding immunity-related proteins, we detected the down-regulated expression of ANKRD22, ICAM1/3, and BIRC5 in the HSAN-VI patients, suggesting changes in cell adhesion and the cytoskeleton, which is in accordance with the expectation that *DST* variants can alter cytoskeletal filament networks (Figure 2E and Supplementary Figure 1). However, we did not observe any change in DST expression by RNAseq or qPCR (Figure 2E).

## Discussion

We report on a Chinese family with HSAN-VI for the first time, revealing an autosomal recessive defect in *DST*. This report further demonstrates a correlation between *DST* and HSAN-VI. Notably, the severities of the sensory and autonomic dysfunctions in the two patients were significantly different, even though they are identical twins who experienced the same conditions until the older brother started to seek treatment. This may be a result of competition for nutrition and space during embryonic development and indicates obvious phenotypic heterogeneity. It has previously been reported that the same *DST* variants lead to family members with different levels of neuronal dysfunction (Manganelli et al., 2017). In addition, the limb phenotypes in one HSAN-VI patient are often different, let alone different individuals. We have also broadened the knowledge of HSAN-VI symptoms, and our results suggest that growth retardation, scoliosis, and corneal ulcerations may be parts of the phenotypes.

Dystonin is a very large cytoskeletal cross-linking protein that interacts with actin and microtubule networks, protein complexes, membrane-bound organelles, and cellular membranes (Ferrier et al., 2014). In humans, *DST* is 500 kb in length and encompasses more than 100 exons (Ferrier et al., 2013). *DST* has four tissue-specific promoters and undergoes many alternative splicing events leading to the expression of numerous isoforms in the central and peripheral nervous systems (dystonin-a), muscle (dystonin-b), and epidermis (dystonin-e) (Kunzli et al., 2016). Promoter-1,−2, and−3 are common promoters active in the nerves and muscle, whereas promoter-e drives tissue-specific dystonin expression in the epidermis (Young et al., 2006). The structure of dystonin-a includes an actin-binding domain (ABD), a plakin domain, spectrin repeats (SRs), and a microtubule-binding domain (MTBD) consisting of an EF-hand pair and a growth arrest-specific 2 (Gas2) domain (Figure 2F) (Suozzi et al., 2012). In contrast to dystonin-a, dystonin-b has two extra plectin repeat domains (PRD2) between the plakin domain and the SRs (Ferrier et al., 2013). Dystonin-e is the smallest, consisting of a plakin domain, a rod domain, and a PRD2 domain (Fine and Mellerio, 2009). The variant (c.3304G>A, p.V1102I) in our patients is located in the plakin domain. This domain plays an important role in cell junctions, and p.V1102I may have effects on its structure and function. The other variant (c.13796G>A, p.R4599H) lies in the spectrin repeats, which give dystonin molecules elasticity to adapt to different membrane structures. The MUpro database (https://www.ics.uci.edu/~baldig/mutation.html) predicted that p.R4599H could decrease protein stability (ΔΔG = −1.074) based on both the value and sign of the energy using a support vector machine (SVM) and sequence information only.

We detected reduced expression of ANKRD22, ICAM1/3, and BIRC5 by RNAseq and qPCR in the proband and his brother. ANKRD22 is a mitochondrial protein and may be associated with schizophrenia (Fromer et al., 2014). DST may affect ANKRD22 to regulate neuronal physiological activities. ICAMs play important roles in cell proliferation, differentiation, motility, and trafficking (Rosette et al., 2005). The change in ICAM1/3 expression suggests an abnormality in cell adhesion. Moreover, DST has been verified can alter cell adhesion and migration (Fortugno et al., 2019). BIRC5 is a member of the inhibitor family of apoptosis and participates in the organization of the central spindle by associating with polymerized microtubules (Kamran et al., 2017). Its reduced expression suggests a reduction in cell proliferation. DST can interact with the cytoskeleton, and its loss of function can inhibit cell proliferation. DST may regulate or interact with ICAMs and BIRC5 to participate in cellular activities and individual development.

The RNAseq and qPCR results did not detect an abnormal expression of DST, which may be because these variants alter the function of the protein, not its expression. Unfortunately, lacking an antibody and patient tissue samples, we cannot further verify our results in regard to alterations of DST expression and function by western blotting.

The c.3304G>A variant has not been reported previously. For the c.13796G>A variant, a frequency of 0.00115 has been reported in the global population. However, in East Asians, the frequency may reach 0.01199. Given this low frequency compared with the frequency in other populations (0.00003 in South Asians, 0.00028 in Latinos, 0.00016 in Europeans, and 0.00132 in Africans) (data from GnomAD), we think that this is the result of a phenomenon similar to a founder effect. It is noted that no carriers have been found to be homozygous for this variation. Given that HSAN-VI is a recessive hereditary disease, the existence of heterozygous carriers does not mean that the c.13796G>A variant is benign. We rechecked the WES results and did not find any variants more credible than the *DST* variants (Supplementary Figure 2, Supplementary Tables 3,4). In particular, we focused on variants in known HSAN-related genes, including *SPTLC1, SPTLC2, ATL1, DNMT1, ATL3, WNK1, FAM134B, KIF1A, SCN9A, IKBKAP, NTRK1, NGFB, DST, SCN11A, PRDM12, CCT5, TECPR2*, and *ZFHX2*. However, no other significant variants were identified (Davidson et al., 2012; Heimer et al., 2016; Yozu et al., 2016).

We summarized all of the *DST* variants previously identified in HSAN-VI patients (Figure 2G) (Edvardson et al., 2012; Manganelli et al., 2017; Fortugno et al., 2019). All of the variants in patients with HSAN-VI involve dystonin-a, which is expressed in nerves, and dystonin-b, which is expressed in muscle. Consistently, all of these patients display neuropathy and myopathy. Our patients also have similar phenotypes. In contrast, variants that affect dystonin-e cause autosomal recessive epidermolysis bullosa simplex (EBS) (Groves et al., 2010). The relationships between *DST* genotypes and their phenotypes require further study.

In summary, the present study identified a novel compound heterozygous *DST* genotype consisting of two missense variants (c.3304G>A, p.V1102I and c.13796G>A, p.R4599H) in identical twins with HSAN-VI. The identification of these variants expands the spectrum of known *DST* variants and further demonstrates that *DST* is associated with HSAN-VI. Our report describes new phenotypes that can assist in the clinical diagnosis of HSAN-VI.

## Data Availability Statement

The original contributions presented in the study are publicly available. This data can be found here: https://www.ncbi.nlm.nih.gov/sra/?term=PRJNA632884.

## Ethics Statement

The Review Board of Xiangya Hospital of the Central South University approved this research. Written informed consent was obtained from the patients and their guardians, and all subjects consented to this study and the publication of the images.

## Author Contributions

J-QH, P-FW, and X-YP collected and provided clinical information. J-YJ, L-LF, and Z-ZY carried out whole exome sequencing and data analysis. L-YZ and J-YT designed experiments and wrote the manuscript.

## Conflict of Interest

The authors declare that the research was conducted in the absence of any commercial or financial relationships that could be construed as a potential conflict of interest.
