# Supplementary figures and images for: Novel Compound Heterozygous DST Variants Causing Hereditary Sensory and Autonomic Neuropathies VI in Twins of a Chinese Family
Source: Front Genet. 2020 May 25;11:492. doi: 10.3389/fgene.2020.00492 (PMC7262964; doi:10.3389/fgene.2020.00492)

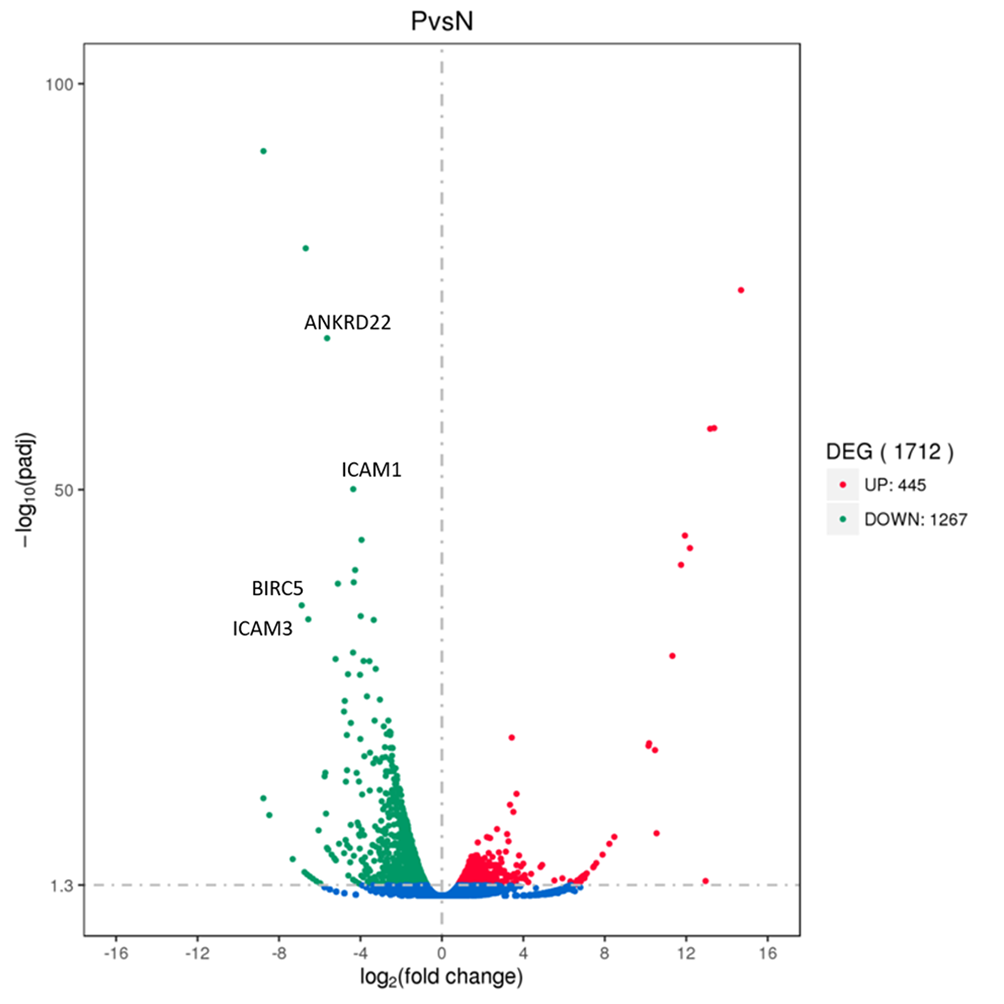

Supplement: Supplementary file 1 [file Image_1.TIF]

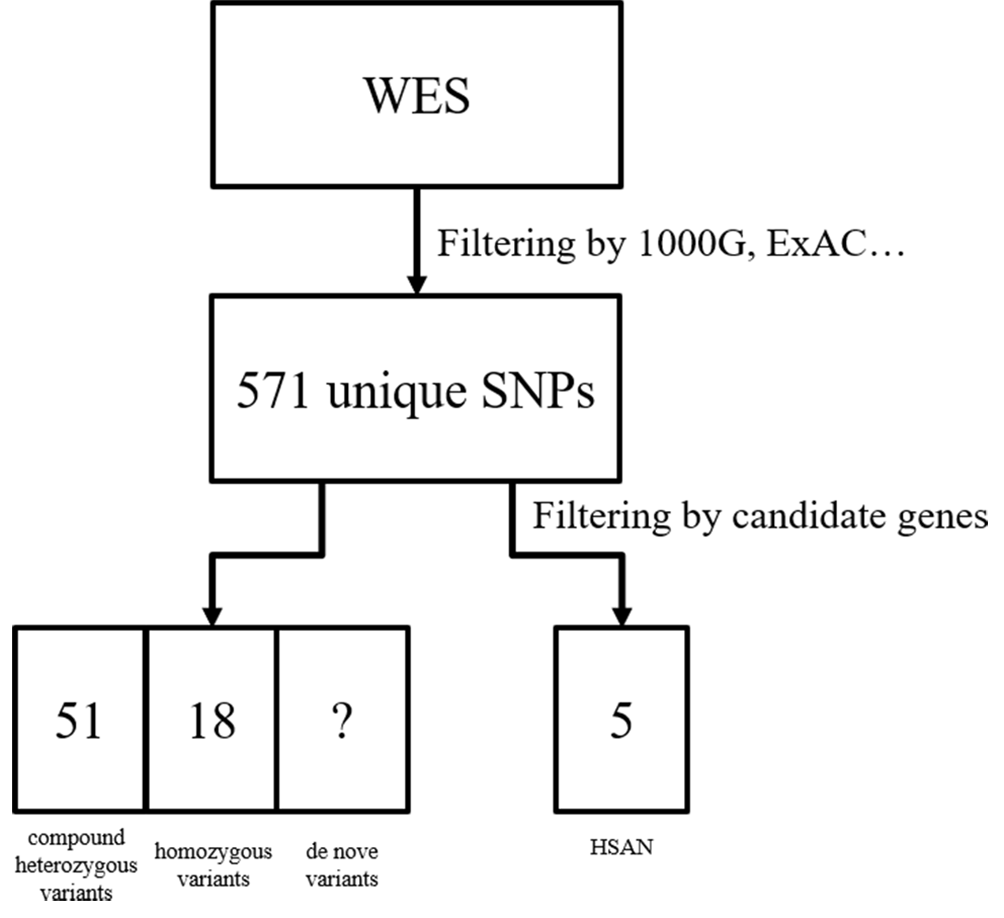

Supplement: Supplementary file 2 [file Image_2.TIF]
