# Supplementary material for: Novel Compound Heterozygous DST Variants Causing Hereditary Sensory and Autonomic Neuropathies VI in Twins of a Chinese Family
Source: Front Genet. 2020 May 25;11:492. doi: 10.3389/fgene.2020.00492 (PMC7262964; doi:10.3389/fgene.2020.00492)
Supplement: Supplementary file 3 [file Table_1.DOC]

**Supplementary Table 1.** Classification of HASN and their causative genes.

| **Subtype** | **OMIM** | **Heredity** | **Gene/Locus** | **Clinical features** | **Age at onset** |
| --- | --- | --- | --- | --- | --- |
| HSAN-IA | 162400 | AD | *SPTLC1* | Loss of pain and temperature sensation; neuropathic pain; ulcerative mutilations; variable distal motor involvement | Adolescence to adulthood |
| HSAN-IB | 608088 | AD | 3p24–p22 | Sensory loss; cough; gastro-oesophageal reflux | Adulthood |
| HASN-IC | 613640 | AD | *SPTLC2* | As for HSAN-IA | Mostly adulthood |
| HSN-ID | 613708 | AD | *ATL1* | Severe distal sensory loss; amyotrophy in the lower limbs; ulcero-mutilating complications; ulcerative mutilations | Adulthood |
| HSN-IE | 614116 | AD | *DNMT1* | Loss of all somatosensory modalities; lancinating pain; ulcerative mutilations; sensorineural deafness; dementia | Adulthood |
| HSN-IF | 615632 | AD | *ATL3* | Sensory loss of the lower limbs; ulcerative mutilations; no autonomic involvement | Adulthood |
| HSAN-IIA | 201300 | AR | *WNK1* | Loss of pain, temperature and touch sensation; detail mutilation; acropathy | Childhood |
| HSAN-IIB | 613115 | AR | *FAM134B* | Impaired nociception of hands and feet; ulcerative mutilations; osteomyelitis; acro-osteolysis | Childhood |
| HSN-IIC | 614213 | AR | *KIF1A* | Impaired position and vibration sensation; ulcerative mutilation; mild motor involvement | Childhood to adolescence |
| HSAN-IID | 243000 | AR | *SCN9A* | Loss of pain and temperature sensation; autonomic disturbances; deafness; hyposmia | Congenital or adolescence |
| HSAN-III | 223900 | AR | *IKBKAP* | Loss of pain and temperature sensation; alacrima; absence of fungiform papillae of the tongue; vasomotor instability; hyperhidrosis | Congenital |
| HSAN-IV | 256800 | AR | *NTRK1* | Loss/Decrease of pain and temperature sensation; anhidrosis; episodic fever; mental retardation; joint deformities | Congenital |
| HSAN-V | 608654 | AR | *NGFB* | Loss/Decrease of pain and temperature sensation; minimal autonomic dysfunction; joint deformities | Congenital |
| HSAN-VI | 614653 | AR | *DST* | Loss/Decrease of pain and temperature sensation; hyperhidrosis; vascular dysregulation; abnormalities of eyes; myopathy; ulcerative mutilations; joint deformities | Congenital |
| HSAN-VII | 615548 | AD | *SCN11A* | Loss of pain sensation; self mutilation; mild muscle weakness and delayed motor development | Congenital |
| HSAN-VIII | 616488 | AR | *PRDM12* | Loss of pain and temperature sensation; ulcerative mutilations; anhidrosis; alacrima; absence of the corneal reflex | Congenital |
| HSN with SPG | 256840 | AR | *CCT5* | Loss of all somatosensory modalities; ulcerative mutilations; spastic paraplegia | Early childhood |
| HSAN with ID | **-** | AR | *TECPR2* | Decreased pain sensitivity; gastro-esophageal reflux; central apneas and areflexia; blood pressure and pulse imbalance; pCO2 accumulation; temperature and osmolarity instability | Congenital |
| Marsili syndrome | 147430 | AD | *ZFHX2* | Pain insensitivity; altered temperature sensation; low sensitivity to capsaicin; decreased or absent sweating; episodic hyperthermia | Childhood |

HSAN = the hereditary sensory and autonomic neuropathy; HSN = the hereditary sensory neuropathy; AD = Autosomal dominant; AR = Autosomal recessive; SPG = Spastic paraplegia; ID = Intellectual disability.
