# Supplementary material for: Novel Compound Heterozygous DST Variants Causing Hereditary Sensory and Autonomic Neuropathies VI in Twins of a Chinese Family
Source: Front Genet. 2020 May 25;11:492. doi: 10.3389/fgene.2020.00492 (PMC7262964; doi:10.3389/fgene.2020.00492)
Supplement: Supplementary file 4 [file Table_2.DOCX]

**Supplementary Table 2.** The nerve conduction study of the proband

| **Motor Nerve Conduction Study** | | | | | | | | | | | | | | | | | | | | | | | | | | | | | | | | | | | | | | | | |
| --- | --- | --- | --- | --- | --- | --- | --- | --- | --- | --- | --- | --- | --- | --- | --- | --- | --- | --- | --- | --- | --- | --- | --- | --- | --- | --- | --- | --- | --- | --- | --- | --- | --- | --- | --- | --- | --- | --- | --- | --- |
| **Site** | **Lat. (ms)** | | | | **N.D.** | | | **Dur. (ms)** | | **Amp.** | | | **N.D.** | | | **Area** | | | | **Stim. (mA)** | **Segment** | | | | | **Dist. (mm)** | | | **Intvl. (ms)** | | | **NCV (m/s)** | | | **CCV (m/s)** | | | **N.D.** | | **Temp** |
| Tibial nerve right | | | | | | | | | | | | | | | |  | | | | | | | | | |  | | |  | | |  | | |  | | |  | |  |
| Ankle | 3.7 | | | |  | | | 5.0 | | 9.0 mV | | |  | | | 8.6 mVms | | | | 49.5 | Ankle | | | | |  | | | 3.7 | | |  | | |  | | |  | |  |
| Popliteal | 10 | | | |  | | | 6.9 | | 7.4 mV | | |  | | | 7.8 mVms | | | | 51.5 | Ankle-Popliteal fossa | | | | | 350 | | | 6.5 | | | 53.8 | | |  | | |  | |  |
| Common peroneal nerves right | | | | | | | | | | | | | | | |  | | | | | | | | | |  | | |  | | |  | | |  | | |  | |  |
| Ankle | 2.8 | | | |  | | | 4.6 | | 1.9 mV | | |  | | | 3.3 mVms | | | | 30.5 | Ankle | | | | |  | | | 2.8 | | |  | | |  | | |  | |  |
| Abv. Fibula | 9.6 | | | |  | | | 6.1 | | 1.3 mV | | |  | | | 3.1 mVms | | | | 37.0 | Ankle-Abv. Fibula | | | | | 310 | | | 6.8 | | | 45.9 | | |  | | |  | |  |
| Tibial nerve left | | | | | | | | | | | | |  | | |  | | | | | | | | | |  | | |  | | |  | | |  | | |  | |  |
| Ankle | 5.5 | | | |  | | | 7.8 | | 8.5 mV | | |  | | | 10.5 mVms | | | | 45 | Ankle | | | | |  | | | 5.5 | | |  | | |  | | |  | |  |
| Popliteal | 12.2 | | | |  | | | 8.9 | | 6.1 mV | | |  | | | 7.9 mVms | | | | 57.5 | Ankle-Popliteal fossa | | | | | 330 | | | 6.8 | | | 48.5 | | |  | | |  | |  |
| Common peroneal nerves left | | | | | | | | | | | | |  | | |  | | | | | | | | | |  | | |  | | |  | | |  | | |  | |  |
| Ankle | 4.6 | | | |  | | | 7.2 | | 4.5 mV | | |  | | | 9.6 mVms | | | | 13.0 | Ankle | | | | |  | | | 4.6 | | |  | | |  | | |  | |  |
| Abv. Fibula | 11.1 | | | |  | | | 7.7 | | 4.3 mV | | |  | | | 9.3 mVms | | | | 26.0 | Ankle-Abv. Fibula | | | | | 305 | | | 6.7 | | | 45.5 | | |  | | |  | |  |
| Median nerve left | | | | | | | | | | | | |  | | |  | | | | | | | | | |  | | |  | | |  | | |  | | |  | |  |
| Wrist | 3.4 | | | |  | | | 6.0 | | 9.7 mV | | |  | | | 21.7 mVms | | | | 16.0 | Wrist | | | | |  | | | 3.4 | | |  | | |  | | |  | |  |
| Elbow | 7.8 | | | |  | | | 6.9 | | 9.0 mV | | |  | | | 21.4 mVms | | | | 25.0 | Wrist-Below | | | | | 215 | | | 4.4 | | | 48.8 | | |  | | |  | |  |
|  |  | | | |  | | |  | |  | | |  | | |  | | | |  | Below-Axilla | | | | |  | | |  | | |  | | |  | | |  | |  |
|  |  | | | |  | | |  | |  | | |  | | |  | | | |  | Axilla-Erb’s | | | | |  | | |  | | |  | | |  | | |  | |  |
| Ulnar nerve left | | | | | | | | | | | | |  | | |  | | | | | | | | | |  | | |  | | |  | | |  | | |  | |  |
| Wrist | 2.2 | | | |  | | | 7.5 | | 10.7 mV | | |  | | | 23.6 mVms | | | | 25.5 | Wrist | | | | |  | | | 2.2 | | |  | | |  | | |  | |  |
| Below | 7.4 | | | |  | | | 8.0 | | 11.0 mV | | |  | | | 27.2 mVms | | | | 32.0 | Wrist-Below | | | | | 200 | | | 3.8 | | | 52.1 | | |  | | |  | |  |
|  |  | | | |  | | |  | |  | | |  | | |  | | | |  | Below-Axilla | | | | |  | | |  | | |  | | |  | | |  | |  |
|  |  | | | |  | | |  | |  | | |  | | |  | | | |  | Axilla-Erb’s | | | | |  | | |  | | |  | | |  | | |  | |  |
| Common peroneal nerves right | | | | | | | | | | | | |  | | |  | | | | | | | | | |  | | |  | | |  | | |  | | |  | |  |
| Wrist | 3.2 | | | |  | | | 6.1 | | 18.8 mV | | |  | | | 40.4 mVms | | | | 26.0 | Wrist | | | | |  | | | 3.2 | | |  | | |  | | |  | |  |
| Elbow |  | | | |  | | |  | |  | | |  | | |  | | | |  | Wrist-Below | | | | | 200 | | | 3.8 | | | 52.1 | | |  | | |  | |  |
|  |  | | | |  | | |  | |  | | |  | | |  | | | |  | Below-Axilla | | | | |  | | |  | | |  | | |  | | |  | |  |
|  |  | | | |  | | |  | |  | | |  | | |  | | | |  | Axilla-Erb’s | | | | |  | | |  | | |  | | |  | | |  | |  |
| Ulnar nerve right | | | | | | | | | | | | |  | | |  | | | | | | | | | |  | | |  | | |  | | |  | | |  | |  |
| Wrist | 2.3 | | | |  | | | 6.0 | | 14.5 mV | | |  | | | 35.0 mVms | | | | 26.5 | Wrist | | | | |  | | | 2.3 | | |  | | |  | | |  | |  |
| Below | 7.4 | | | |  | | | 6.8 | | 13.9 mV | | |  | | | 35.2 mVms | | | | 36.0 | Wrist-Below | | | | | 270 | | | 5.0 | | | 53.6 | | |  | | |  | |  |
|  |  | | | |  | | |  | |  | | |  | | |  | | | |  | Below-Axilla | | | | |  | | |  | | |  | | |  | | |  | |  |
|  |  | | | |  | | |  | |  | | |  | | |  | | | |  | Axilla-Erb’s | | | | |  | | |  | | |  | | |  | | |  | |  |
| Radial nerve left | | | | | | | | | | | | |  | | |  | | | | | | | | | |  | | |  | | |  | | |  | | |  | |  |
| Elbow | 2.0 | | | |  | | | 10.4 | | 13.5 mV | | |  | | | 44.7 mVms | | | | 30.5 | Elbow-Radial nerve groove | | | | |  | | | 1.7 | | |  | | |  | | |  | |  |
| Radial nerve groove | 3.7 | | | |  | | | 9.3 | | 10.3 mV | | |  | | | 35.1 mVms | | | | 41.0 | Elbow (right) | | | | |  | | | 2.3 | | |  | | |  | | |  | |  |
| Radial nerve right | | | | | | | | | | | | |  | | |  | | | | | | | | | |  | | |  | | |  | | |  | | |  | |  |
| Elbow | 2.3 | | | |  | | | 9.4 | | 15.7 mV | | |  | | | 50.3 mVms | | | | 27.0 |  | | | | |  | | |  | | |  | | |  | | |  | |  |
| Radial nerve groove | 3.9 | | | |  | | | 8.7 | | 15.2 mV | | |  | | | 47.8 mVms | | | | 27.0 |  | | | | |  | | |  | | |  | | |  | | |  | |  |
| Musculocutaneous nerve | | | | | | | | | | | | |  | | |  | | | | | | | | | |  | | |  | | |  | | |  | | |  | |  |
| Left | 3.9 | | | |  | | | 25.6 | | 9.3 mV | | |  | | | 65.0 mVms | | | | 58.5 | Left | | | | |  | | |  | | |  | | |  | | |  | |  |
| Right | 3.8 | | | |  | | | 18.2 | | 13.5 mV | | |  | | | 52.6 mVms | | | | 68.5 |  | | | | |  | | |  | | |  | | |  | | |  | |  |
| Axillary nerve | | | | | | | | | | | | |  | | |  | | | | | | | | | |  | | |  | | |  | | |  | | |  | |  |
| Left | 1.8 | | | |  | | | 8.8 | | 23.8 mV | | |  | | | 82.2 mVms | | | | 45.0 |  | | | | |  | | |  | | |  | | |  | | |  | |  |
| Right | 2.3 | | | |  | | | 9.1 | | 21.8 mV | | |  | | | 80.9 mVms | | | | 45.5 |  | | | | |  | | |  | | |  | | |  | | |  | |  |
| **Sensory Nerve Conduction Study** | | | | | | | | | | | | | | | | | | | | | | | | | | | | | | | | | | | | | | | | |
| **Site** | **Lat.1 (ms)** | | | | **N.D.** | | | **Lat.2 (ms)** | | **Amp.** | | | **N.D.** | | | **Area** | | | | **Stim. (mA)** | **Segment** | | | | | **Dist. (mm)** | | | **Intvl. (ms)** | | | **NCV (m/s)** | | | **CCV. (m/s)** | | | **N.D.** | | **Temp** |
| Sural nerve left | | | | | | | | | | | | |  | | |  | | | | | | | | | |  | | |  | | |  | | |  | | |  | |  |
| Sural |  | | | |  | | |  | |  | | |  | | |  | | | | 27.5 | Sural | | | | | 100 | | |  | | |  | | |  | | |  | |  |
|  |  | | | |  | | |  | |  | | |  | | |  | | | | 27.5 |  | | | | |  | | |  | | |  | | |  | | |  | |  |
| Sural nerve right | | | | | | | | | | | | |  | | |  | | | | | | | | | |  | | |  | | |  | | |  | | |  | |  |
| Sural |  | | | |  | | |  | |  | | |  | | |  | | | | 27.5 | Sural | | | | | 125 | | |  | | |  | | |  | | |  | |  |
| Median nerve left | | | | | | | | | | | | |  | | |  | | | | | | | | | |  | | |  | | |  | | |  | | |  | |  |
| Wrist-Dig3 | | | | |  | | |  | |  | | |  | | |  | | | | 23.5 | Wrist-Dig3 | | | | |  | | |  | | |  | | |  | | |  | |  |
|  |  | | | |  | | |  | |  | | |  | | |  | | | |  | Wrist-Dig1 | | | | |  | | |  | | |  | | |  | | |  | |  |
| Ulnar nerve left | | | | | | | | | | | | |  | | |  | | | | | | | | | |  | | |  | | |  | | |  | | |  | |  |
| Wrist |  | | | |  | | |  | |  | | |  | | |  | | | | 13.0 | Wrist | | | | |  | | |  | | |  | | |  | | |  | |  |
| Median nerve right | | | | | | | | | | | | |  | | |  | | | | | | | | | |  | | |  | | |  | | |  | | |  | |  |
| Wrist-Dig3 | | | | |  | | |  | |  | | |  | | |  | | | | 33.0 | Wrist-Dig3 | | | | |  | | |  | | |  | | |  | | |  | |  |
|  |  | | | |  | | |  | |  | | |  | | |  | | | |  | Wrist-Dig1 | | | | |  | | |  | | |  | | |  | | |  | |  |
| Ulnar nerve right | | | | | | | | | | | | |  | | |  | | | | | | | | | |  | | |  | | |  | | |  | | |  | |  |
| Wrist |  | | | |  | | |  | |  | | |  | | |  | | | | 23.5 | Wrist | | | | |  | | |  | | |  | | |  | | |  | |  |
| Radial nerve | | | | | | | | | | | | |  | | |  | | | | | | | | | |  | | |  | | |  | | |  | | |  | |  |
| Left |  | | | |  | | |  | |  | | |  | | |  | | | | 22.5 | Left | | | | | 115 | | |  | | |  | | |  | | |  | |  |
| Right | 2.2 | | | |  | | | 3.8 | | 1.0 μV | | |  | | | 1.1μVms | | | | 22.5 | Right | | | | | 110 | | | 2.2 | | | 49.1 | | |  | | |  | |  |
| **F-wave** | | | | | | | | | | | | | | | | | | | | | | | | | | | | | | | | | | | | | | | | |
| **Nerve** | | **Side** | | | | **Stim. Site** | | | | | | **F-Lat.** | | | **F-Lat. N.D.** | | | | **M Lat.** | | | **F-M Lat.** | | | **F-Occurr.** | | | | | **Distance** | | | | | | **FWCV** | | | **N.D.** | |
| Tibial nerve | | Right | | | |  | | | | | | 41.4 ms | | |  | | | | 0.0 ms | | | 41.4 ms | | | ? | | | | |  | | | | | | *** | | |  | |
| Tibial nerve | | Left | | | |  | | | | | | 46.8 ms | | |  | | | | 0.0 ms | | | 46.8 ms | | | ? | | | | |  | | | | | | *** | | |  | |
| Median nerve | | Right | | | | Wrist | | | | | | 26.0 ms | | |  | | | | 0.0 ms | | | 26.0 ms | | | ? | | | | | 730 mm | | | | | | 58.5 m/s | | |  | |
| Median nerve | | Left | | | | Wrist | | | | | | 23.9 ms | | |  | | | | 0.0 ms | | | 23.9 ms | | | ? | | | | | 730 mm | | | | | | 63.8 m/s | | |  | |
| **H-reflex** | | | | | | | | | | | | | | | | | | | | | | | | | | | | | | | | | | | | | | | | |
| **Nerve** | | | **Side** | | | | | | **Stim. Site** | | | | **Rin. Site** | | | | **M-Latency** | | | | | | **M-Amp. Max** | | | | | **H-Latency** | | | | | **H-Amp. Max** | | | | | **H/M Ratio** | | |
| Tibial nerve | | | Left | | | | | |  | | | |  | | | |  | | | | | | 0.3 mV | | | | |  | | | | |  | | | | |  | | |
| Tibial nerve | | | Right | | | | | |  | | | |  | | | |  | | | | | | 0.6 mV | | | | |  | | | | |  | | | | |  | | |
| **EMG Findings Summary** | | | | | | | | | | | | | | | | | | | | | | | | | | | | | | | | | | | | | | | | |
| **Muscle** | | | | **Side** | | | **Insert. potential** | | | | **Fibrillation** | | | **Positive sharp wave** | | | | **Fasciculation** | | | | | | **Other potential** | | | **Polyphase wave** | | | | **Amp.** | | | **Time** | | | **Recruitment order** | | | |
| Medial vastus muscle | | | | Right | | | Normal | | | | Null | | | Null | | | | Null | | | | | | Null | | | Normal | | | | Normal | | | Normal | | | Normal | | | |
| Medial head of gastrocnemius | | | | Left | | | Normal | | | | Null | | | Null | | | | Null | | | | | | Null | | | Normal | | | | Normal | | | Normal | | | Normal | | | |
| Tibialis anterior | | | | Left | | | Normal | | | | Null | | | Null | | | | Null | | | | | | Null | | | Normal | | | | Normal | | | Normal | | | Normal | | | |
| Medial vastus muscle | | | | Left | | | Normal | | | | Null | | | Null | | | | Null | | | | | | Null | | | Normal | | | | Normal | | | Normal | | | Normal | | | |
| Deltoid muscle | | | | Right | | | Normal | | | | Null | | | Null | | | | Null | | | | | | Null | | | Normal | | | | Normal | | | Normal | | | Normal | | | |
| Biceps brachii | | | | Right | | | Normal | | | | Null | | | Null | | | | Null | | | | | | Null | | | Normal | | | | Normal | | | Normal | | | Normal | | | |
| Abductor pollicis brevis | | | | Right | | | Normal | | | | Null | | | Null | | | | Null | | | | | | Null | | | Normal | | | | Normal | | | Normal | | | Normal | | | |
| Abductor pollicis brevis | | | | Left | | | Normal | | | | Null | | | Null | | | | Null | | | | | | Null | | | Normal | | | | Normal | | | Normal | | | Lower | | | |
| Abductor digiti minimi | | | | Left | | | Normal | | | | Null | | | Null | | | | Null | | | | | | Null | | | Normal | | | | Normal | | | Normal | | | Lower | | | |
