# Supplementary material for: Novel Compound Heterozygous DST Variants Causing Hereditary Sensory and Autonomic Neuropathies VI in Twins of a Chinese Family
Source: Front Genet. 2020 May 25;11:492. doi: 10.3389/fgene.2020.00492 (PMC7262964; doi:10.3389/fgene.2020.00492)
Supplement: Supplementary file 5 [file Table_3.DOCX]

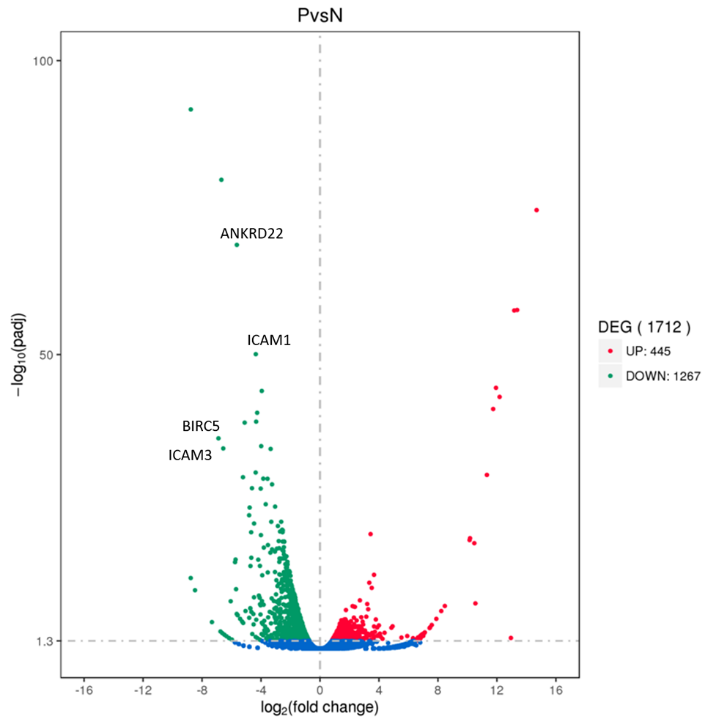


**Fig.S1.** The volcano plot of RNAseq. P indicates patients (II:1 and II:2); N indicates the normal (I:2).


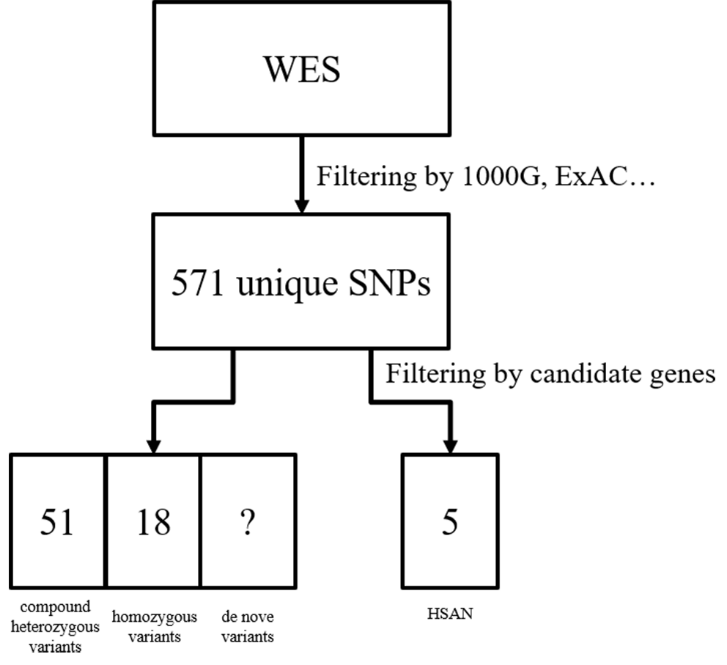


**Fig.S2.** Schematic diagram of rechecking WES results of the proband.
