# Supplementary material for: Novel Compound Heterozygous DST Variants Causing Hereditary Sensory and Autonomic Neuropathies VI in Twins of a Chinese Family
Source: Front Genet. 2020 May 25;11:492. doi: 10.3389/fgene.2020.00492 (PMC7262964; doi:10.3389/fgene.2020.00492)
Supplement: Supplementary file 6 [file Table_4.DOCX]

**Supplementary Table 3.** The compound heterozygous variants in the proband^#^

| **Gene** | **Variant** | **MutationTaster** | **PolyPhen-2** | **SIFT** | **OMIM clinical**  **phenotype** | **ToppGene function** | **American College**  **of Medical Genetics**  **classification** |
| --- | --- | --- | --- | --- | --- | --- | --- |
| *ANKRD1* | NM_152763: c.2167+1G>A | D (1.000) | P (0.808) | D (0.012) | - | titin binding | PM4 |
| *ANKRD1* | NM_152763: c.1675G>T, p.G559C | N (1.000) | P (0.712) | D (0.022) |  |  | PP3 |
| *ANKLE1* | NM_001278444: c.1922_1933del, p.641_645del | - | - | - | - | - | PP3 |
| *ANKLE1* | NM_001278444: c.1924_1933del, p.C642fs | D (1.000) | - | - |  |  | PM4 |
| *ARAP2* | NM_015230: c.2778G>T, p.L926F | D (0.958) | P (0.745) | T (0.071) | - | phosphatidylinositol-3,4,5-trisphosphate binding | PM2 |
| *ARAP2* | NM_015230: c.1493G>A, p.R498H | D (0.847) | D (0.983) | T (0.128) |  |  | PP3 |
| *BAIAP2L1* | NM_018842: c.487-7_8insATTATT | N (1.000) | - | - | - | cytoskeletal adaptor activity | BS4 |
| *BAIAP2L1* | NM_018842: c.215-8del | N (1.000) | - | - |  |  | BP4 |
| *CELSR2* | NM_001408: c.1792G>A, p.V598M | D (0.974) | B (0.210) | T (0.069) | - | cerebrospinal fluid secretion | PP3 |
| *CELSR2* | NM_001408: c.2084C>T, p.T695M | D (1.000) | D (0.993) | D (0.004) |  |  | PP3 |
| *COL6A6* | NM_001102608: c.574C>T, p.L192F | N (0.892) | P (0.867) | D (0.005) | - | collagen catabolic process | PP3 |
| *COL6A6* | NM_001102608: c.2356C>T, p.R786C | N (1.000) | B (0.264) | T (0.067) |  |  | BP6 |
| *DCHS1* | NM_003737: c.395G>T, p.R132L | N (0.635) | D (0.979) | T (0.271) | AD, Mitral valve prolapse 2/AR, Van Maldergem syndrome 1 | calcium ion binding | PM2 |
| *DCHS1* | NM_003737: c.97_99del, p.L33del | N (0.851) | - | - |  |  | PP3 |
| *DNAH3* | NM_017539: c.10827-10G>A | N (1.000) | - | - | - | calcium ion binding | BP4 |
| *DNAH3* | NM_017539: c.6827G>A, p.R2276Q | D (1.000) | D (1.000) | D (0.000) |  |  | BS4 |
| *DSPP* | NM_014208: c.932A>G, p.E311G | N (1.000) | P (0.889) | - | AD, Deafness, autosomal dominant 39, with dentinogenesis /AD, Dentin dysplasia, type II/AD, Dentinogenesis imperfecta, Shields type II/AD Dentinogenesis imperfecta, Shields type III | collagen binding | PP3 |
| *DSPP* | NM_014208: c.2017G>A, p.D673N | N (1.000) | B (0.322) | - |  |  | PP3 |
| *DST* | NM_001144769: c.13796G>A, p.R4599H | D (1.000) | D (1.000) | T (0.136) | AR, Neuropathy, hereditary sensory and autonomic, type VI/AR, Epidermolysis bullosa simplex, autosomal recessive 2 | microtubule plus-end binding | PP4 |
| *DST* | NM_001144769: c.3304G>A, p.V1102I | D (1.000) | D (0.996) | D (0.000) |  |  | PM2 |
| *FCGBP* | NM_003890: c.9511C>T, p.P3171S | N (1.000) | D (0.999) | T (0.296) | - | extracellular matrix | PP3 |
| *FCGBP* | NM_003890: c.8008G>A, p.G2670R | D (0.993) | D (1.000) | D (0.012) |  |  | PP3 |
| *KDM6B* | NM_001080424: c.752_757del, p.251_253del | N (1.000) | - | - | - | histone demethylase activity (H3-K27 specific) | PM2 |
| *KDM6B* | NM_001080424: c.768_769insCCACCC, p.P256delinsPPP | N (1.000) | - | - |  |  | PP3 |
| *KDM6B* | NM_001080424: c.774_775insCCACCC, p.P258delinsPPP | N (1.000) | - | - |  |  | PM2 |
| *KIF24* | NM_194313: c.2971C>T, p.H991Y | N (1.000) | B (0.188) | D (0.000) | - | microtubule depolymerization | PP3 |
| *KIF24* | NM_194313: c.881C>T, p.T294I | D (1.000) | P (0.497) | D (0.045) |  |  | PP3 |
| *MUC19* | NC_000012.11: g.40876719G>A | - | - | - | - | O-glycan processing | - |
| *MUC19* | NC_000012.11: g.40876727G>A | - | - | - |  |  | - |
| *MUC21* | NM_001010909: c.404C>T, p.A135V | D (0.933) | P (0.719) | T (0.280) | - | negative regulation of cell-substrate adhesion | PP3 |
| *MUC21* | NM_001010909: c.968C>T, p.T323I | N (1.000) | B (0.225) | T (0.170) |  |  | BP6 |
| *PABPC3* | NM_030979: c.440C>T, p.T147I | D (0.999) | P (0.749) | D (0.000) | - | poly(A) binding | PP3 |
| *PABPC3* | NM_030979: c.1406G>A, p.R469Q | D (0.991) | B (0.002) | T (0.239) |  |  | PP3 |
| *PER3* | NM_016831: c.2756C>T, p.S919L | N (1.000) | D (0.997) | D (0.000) | AD, ?Advanced sleep phase syndrome, familial, 3 | ubiquitin protein ligase binding | BS4 |
| *PER3* | NM_016831: c.976+10_11insA | N (1.000) | - | - |  |  | BP4 |
| *PRIM2* | NM_000947: c.794A>T, p.Q265L | - | - | - | - | ATP coenzyme F420 adenylyltransferase activity | PP3 |
| *PRIM2* | NM_000947: c.967A>G, p.T265A | - | - | - |  |  | PP3 |
| *SCAF11* | NM_004719: c.3781G>C, p.V1261L | N (0.842) | B (0.376) | T (0.103) | - | spliceosomal complex assembly | BP6 |
| *SCAF11* | NM_004719: c.2648C>T, p.P883L | D (1.000) | D (0.992) | D (0.000) |  |  | PP3 |
| *SVEP1* | NM_153366: c.1813G>T, p.V605F | D (0.993) | D (0.999) | D (0.004) | - | - | PP3 |
| *SVEP1* | NM_153366: c.1196A>G, p.N399S | D (0.911) | P (0.725) | T (0.071) |  |  | PP3 |
| *TBC1D1* | NM_001253912: c.1429G>A, p.A477T | D (0.995) | P (0.784) | T (0.185) | - | Rab GTPase binding | PP3 |
| *TBC1D1* | NM_001253912: c.2169_2170del, p.T723fs | D (1.000) | - | - |  |  | PM4 |
| *TTN* | NM_001267550: c.102271C>T, p.R34091W | D (0..999) | D (0.946) | D (0.001) | Cardiomyopathy, dilated, 1G/AD, Cardiomyopathy, familial hypertrophic, 9/AR, Muscular dystrophy, limb-girdle, autosomal recessive 10/AD Myopathy, myofibrillar, 9, with early respiratory failure/AD, Salih myopathy/AD, Tibial muscular dystrophy, tardive | telethonin binding | PM5 |
| *TTN* | NM_001267550: c.18407G>A, p.R6136Q | D (1.000) | B (0.003) | T (0.188) |  |  | PP3 |
| *VPS13C* | NM_020821: c.7861G>A, p.V2621I | D (1.000) | B (0.396) | - | AR, arkinson disease 23, autosomal recessive, early onset | negative regulation of parkin-mediated stimulation of mitophagy in response to mitochondrial depolarization | BS4 |
| *VPS13C* | NM_020821: c.1291-4A>G | N (1.000) | - | - |  |  | BP4 |
| *XIRP2* | NM_152381: c.862G>T, p.A288S | N (1.000) | B (0.200) | D (0.008) | - | alpha-actinin binding | PP3 |
| *XIRP2* | NM_152381: c.3001G>A, p.V1001I | D (1.000) | P (0.536) | D (0.024) |  |  | PP3 |
| *ZFHX2* | NM_033400: c.3676C>T, p.P1226S | D (1.000) | B (0.435) | T (0.378) | AD, ?Marsili syndrome | adult behavior | PM2 |
| *ZFHX2* | NM_033400: c.3684_3689del, p.1228_1230del | N (1.000) | - | - |  |  | PM2 |

^#^, at least 2 variants of the same gene were identified by WES, without verification by Sanger sequencing, so they may be not compound heterozygous.
