# Supplementary material for: Novel Compound Heterozygous DST Variants Causing Hereditary Sensory and Autonomic Neuropathies VI in Twins of a Chinese Family
Source: Front Genet. 2020 May 25;11:492. doi: 10.3389/fgene.2020.00492 (PMC7262964; doi:10.3389/fgene.2020.00492)
Supplement: Supplementary file 7 [file Table_5.DOCX]

**Supplementary Table 4.** The homozygous variants in the proband.

| **Gene** | **Variant** | **MutationTaster** | **PolyPhen-2** | **SIFT** | **OMIM clinical**  **phenotype** | **ToppGene function** | **American College**  **of Medical Genetics**  **classification** |
| --- | --- | --- | --- | --- | --- | --- | --- |
| *ANKRD1* | NM_152763: c.346-10_13del | N (1.000) | - | - | - | titin binding | BP4 |
| *ANKRD1* | NM_152763: c.346-10_17del | N (1.000) | - | - |  |  | BP4 |
| *CASKIN1* | NM_020764: c.4056_4064del, p.1352_1355del | N (1.000) | - | - | - | protein domain specific binding | PP3 |
| *DDX53* | NM_182699: c.640C>T, p.R214C | D (1.000) | D (1.000) | D (0.039) | - | RNA-dependent ATPase activity | PP3 |
| *DMD* | NM_004006: c.11015-4_6del | N (1.000) | - | - | XLR, Duchenne muscular dystrophy | dystroglycan binding | BP4 |
| *DPP4* | NM_001935: c.1177-7_9del | N (1.000) | - | - | - | dipeptidyl-peptidase activity | BP4 |
| *F8* | NM_000132: c.4931C>T, p.T1644I | N (1.000) | B (0.125) | D (0.002) | XLR, Hemophilia A | copper ion binding | PP3 |
| *FAM83G* | NM_001039999: c.2455_2456insCCC, p.D819delinsAH | N (1.000) | - | - | - | BMP signaling pathway | PP3 |
| *GAB3* | NM_080612: c.1354C>G, p.P452A | D (0.648) | B (0.430) | D (0.005) | - | macrophage differentiation | PP3 |
| *HAUS7* | NM_017518: c.961-4_7del | N (1.000) | -- | - | - | thioesterase binding | BP4 |
| *NBPF9* | NM_001277444: c.117_117insAC, p.C40fs | D (1.000) | - | - | - | - | PM4 |
| *PA2G4* | NM_006191: c.217+2_3insAA | N (1.000) | - | - | - | ubiquitin protein ligase binding | BP4 |
| *PASD1* | NM_173493: c.2243A>G, p.Y748C | N (1.000) | B (0.061) | D (0.000) | - | transcription coactivator binding | PP3 |
| *PPM1E* | NM_014906: c.126_127insCCCGAG, p.E42delinsEPE | N (1.000) | - | - | - | protein serine/threonine phosphatase activity | PP3 |
| *SIRPB1* | NM_001135844: c.392_393del, p.V131fs | D (1.000) | - | - | - | positive regulation of phagocytosis | PM4 |
| *TBL1X* | NM_005647: c.480_481insGCGGCG, p.S160delinsSAA | N (1.000) | - | - | - | beta-catenin binding | PP3 |
| *TTLL3* | NM_001025930: c.371_372insTGG, p.D124delinsDG | N (1.000) | - | - | - | protein-glycine ligase activity, elongating | PP3 |
| *ZFYVE19* | NM_001077268: c.110_111insGTGGG, p.V37fs | D (1.000) | - | - | - | phosphatidylinositol-3-phosphate binding | PM4 |
